# Supplementary material for: Algorithmic versus human surveillance leads to lower perceptions of autonomy and increased resistance
Source: Commun Psychol. 2024 Jun 6;2:53. doi: 10.1038/s44271-024-00102-8 (PMC11332184; doi:10.1038/s44271-024-00102-8)

Supplementary Online Materials

Algorithmic Versus Human Surveillance Leads to Lower Perceptions of Autonomy and Increased Resistance

Rachel Schlund and Emily M. Zitek

Department of Organizational Behavior, Cornell University, Ithaca, NY, USA

Supplementary Note 1

In our pre-registration, we made an error in that we referred to the measure of resistance behaviors in this study as a measure of intentions to quit, but this was just one item on the 4-item scale. We report the results from the full scale, as intended, in the main paper.

Supplementary Note 2

In this section, we report the results from our main dependent variables analyzed in an additional way as a robustness check.

In Study 2, although participants completed the dependent variable measures individually, it is possible that characteristics of their original group could have affected their responses. To examine this possibility, we calculated the ICCs, which ranged from .00 to .19, indicating some small group effects. To confirm that the results were the same when accounting for the group effects, we reran the analyses using mixed models with a random intercept for group ID and fixed effects for group size and surveillance source. The results remained the same as what was reported in the paper. Specifically, participants put under algorithmic (v. human) surveillance engaged in more criticism and performed worse (see Supplementary Tables 1 & 2).

Supplementary Table 1

Results from a mixed model predicting criticism (N = 157).

	b (*se*)	*Z*	*p*
Intercept	-0.95 (1.15)	-0.83	.409
Algorithmic (coded 1) v. Human Surveillance (coded -1)	1.98 (0.54)	3.70	.000
Group Size	-0.40 (0.25)	-1.58	.114

Note. Pseudo R^2 = 0.23 (fixed effects), 0.23 (total), ICC = 0.00

Supplementary Table 2

Results from a mixed model predicting individual-level productivity (N = 157).

	b (*se*)	*t*	*p*
Intercept	11.76 (2.42)	4.86	.000
Algorithmic (coded 1) v. Human Surveillance (coded -1)	-2.10 (0.92)	-2.27	.031
Group Size	-0.77 (0.54)	-1.43	.161

Note. Pseudo R^2 = 0.08 (fixed effects), 0.25 (total), ICC = 0.19

Supplementary Note 3

We also included two additional dependent variables in Study 2 that were not reported in the main paper. To measure participants' perceived privacy invasion, we used a pre-established 11-item scale (Alge, 2001). Example items include: "I feel like the manner in which I am evaluated is an invasion of my privacy" and "I feel that the methods used to monitor my performance are invasive." Participants who were monitored and evaluated by algorithmic surveillance perceived a greater invasion of their privacy (M = 3.43, SD = 1.13) than participants who were monitored and evaluated by human surveillance (M = 2.68, SD = 0.91), $t(155) = -4.57$, $p < .001$, $d = -0.73$.

Further, a mixed model predicting perceived privacy invasion from monitoring and evaluation source (algorithmic v. human surveillance) with a random intercept for group ID and group size as a fixed effect control revealed the same pattern of results (see Supplementary Table 3).

Supplementary Table 3

Results from a mixed model predicting perceived privacy invasion (N = 157).

	b (*se*)	*t*	*p*
Intercept	2.98 (0.49)	6.08	.000
Algorithmic (coded 1) v. Human Surveillance (coded -1)	0.77 (0.18)	4.31	.000
Group Size	-0.07 (0.11)	-0.62	.536

Note. Pseudo R^2 = 0.12 (fixed effects), 0.16 (total), ICC = 0.04

Supplementary Note 4

Due to the design of the Study 2, we were able to collect performance data at the group level during the first brainstorming task participants completed. Given the low number of observations at the group level (*n* = 36), we do not want to highlight these results to a great extent, but we report them here: The difference in group productivity between the algorithmic surveillance and human surveillance conditions failed to reach significance (*M* = 25.72, *SD* = 16.61 v. *M* = 28.72, *SD* = 11.21), *t*(34) = 0.64, *p* = .529, *d* = 0.21.

Supplementary Note 5

As in the previous study, below we report an alternative analysis of our dependent variables.

As in Study 2, we found some small group effects in Study 3 (ICCs from .00 to .10), and we wanted to check to be sure our results held when accounting for these small group effects. We again ran mixed models, this time predicting perceived autonomy, criticism, and productivity from surveillance source (algorithmic vs. human), with a random intercept for group ID and group size as a fixed effect control. The results from the mixed models were very similar to the results of the pre-registered analyses reported in the main-text, with the only change being that

the effect of surveillance source on individual performance was now marginally significant (p = .066). See Supplementary Tables 4–6.

Supplementary Table 4

Results from a mixed model predicting perceived autonomy (N = 117).

	b (*se*)	*t*	*p*
Intercept	4.44 (0.31)	14.46	.000
Algorithmic (coded 1) v. Human Surveillance (coded -1)	-0.34 (0.10)	-3.42	.003
Group Size	-0.04 (0.07)	-0.62	.542

Note. Pseudo R^2 = 0.11 (fixed effects), 0.14 (total), ICC = 0.03

Supplementary Table 5

Results from a mixed model predicting criticism (N = 117).

	b (*se*)	*t*	*p*
Intercept	0.10 (0.07)	1.43	.161
Algorithmic (coded 1) v. Human Surveillance (coded -1)	0.06 (0.02)	2.77	.010
Group Size	-0.01 (0.01)	-0.60	.554

Note. Pseudo R^2 = 0.06 (fixed effects), 0.06 (total), ICC = 0.00; Results of a linear mixed model are reported as a generalized linear mixed model would not converge (seemingly due to the lack of criticism in the human surveillance condition).

Supplementary Table 6

Results from a mixed model predicting individual-level productivity (N = 117).

	b (*se*)	*t*	*p*
Intercept	7.05 (1.17)	6.01	.000
Algorithmic (coded 1) v. Human Surveillance (coded -1)	-0.75 (0.39)	-1.91	.066
Group Size	-0.02 (0.26)	-0.09	.929

Note. Pseudo R^2 = 0.04 (fixed effects), 0.14 (total), ICC = 0.10

Supplementary Note 6

In Study 3, we measured perceived privacy invasion in the same way as in Study 2. Participants who were monitored and evaluated by algorithmic surveillance perceived a greater invasion of their privacy (*M* = 3.92, *SD* = 1.06) than participants who were monitored and evaluated by human surveillance (*M* = 2.91, *SD* = 0.98), $t(115) = -5.35$, $p < .001$, d =-0.99. Further, a mixed model predicting perceived privacy from monitoring and evaluation source (human surveillance v. algorithmic surveillance) with a random intercept for group ID and with group size as a fixed effect control revealed the same pattern of results (see Supplementary Table 7).

Supplementary Table 7

Results from a mixed model predicting perceived privacy invasion (N = 117).

	b (*se*)	*t*	*p*
Intercept	3.37 (0.30)	11.20	.000
Algorithmic (coded 1) v. Human Surveillance (coded -1)	0.51 (0.10)	5.23	.000
Group Size	0.01 (0.07)	0.15	.884

Note. Pseudo R^2 = 0.20 (fixed effects), 0.20 (total), ICC = 0.00

Supplementary Note 7

Given the low number of observations at the group level in Study 3 (*n* = 30), we do not want to highlight these performance effects to a great extent, but we report them here: Participants who were monitored and evaluated by algorithmic surveillance came up with fewer responses at the group level (*M* = 24.33, *SD* = 17.09) than the participants who were monitored and evaluated by human surveillance (*M* = 27.73, *SD* = 14.94); however, as in Study 2, this difference failed to reach significance, $t(28) = 0.58$, p =.567, d =0.21.

Supplementary Figure 1

Perceived Autonomy by Condition in Study 1.

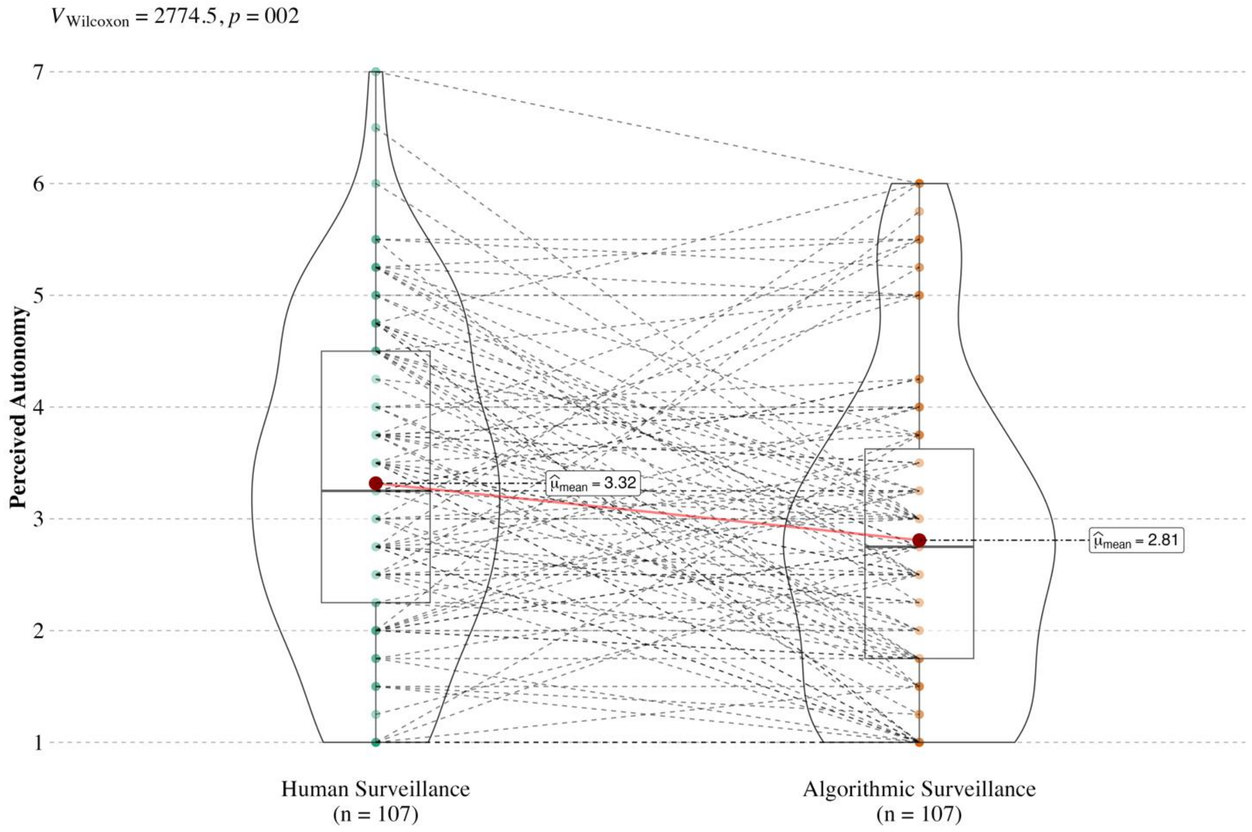

Supplementary Figure 2

Resistance Intentions by Condition in Study 1.

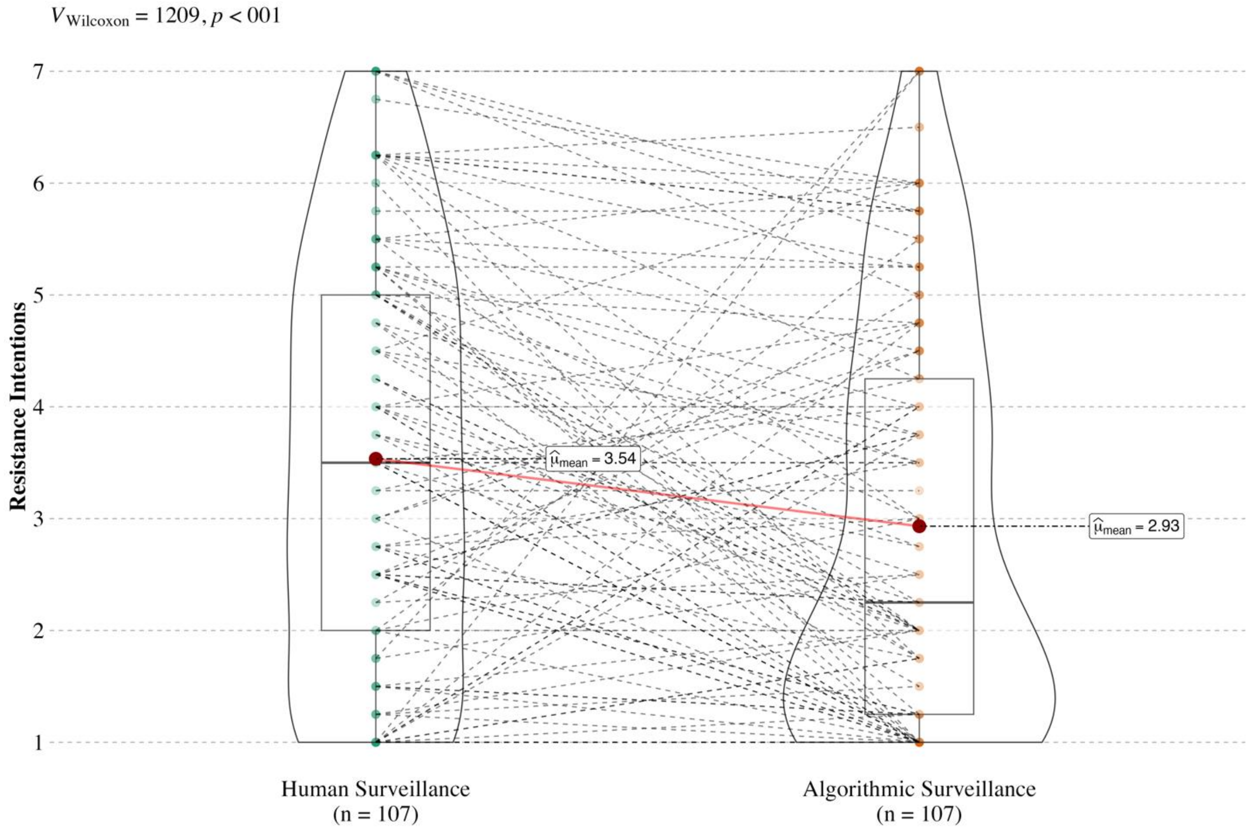

Supplementary Note 8

As seen in Supplementary Figures 3 and 4, the performance data in Studies 2 and 3 was positively skewed. Thus, in addition to the Mann-Whitney U test, we ran further robustness checks. Specifically, we reanalyzed the data after removing outliers, and we also reanalyzed the data after performing natural log and square root transformations of performance. These additional analyses all revealed the same pattern of results as reported in the main paper.

Supplementary Figure 3

Performance by Condition in Study 2.

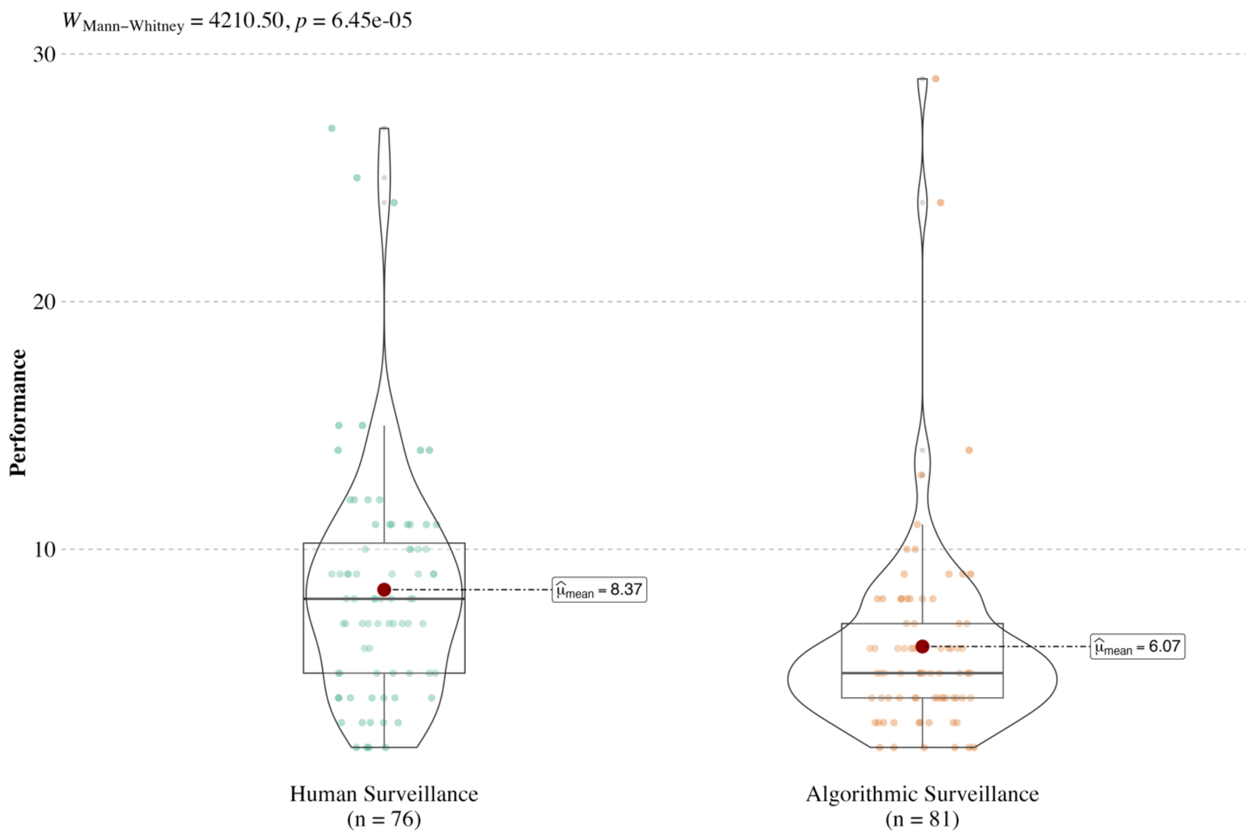

Supplementary Figure 4

Performance by Condition in Study 3.

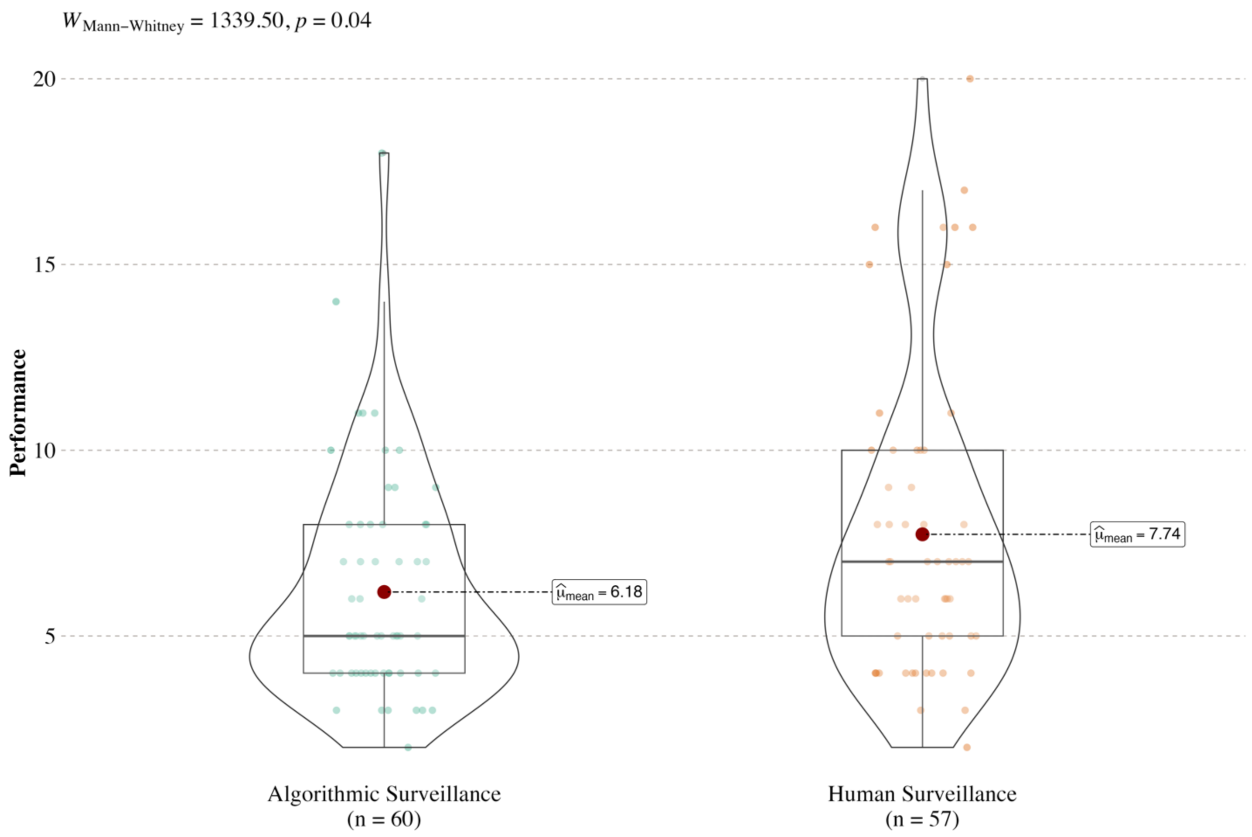

Supplementary Figure 5

Perceived Autonomy by Condition in Study 3.

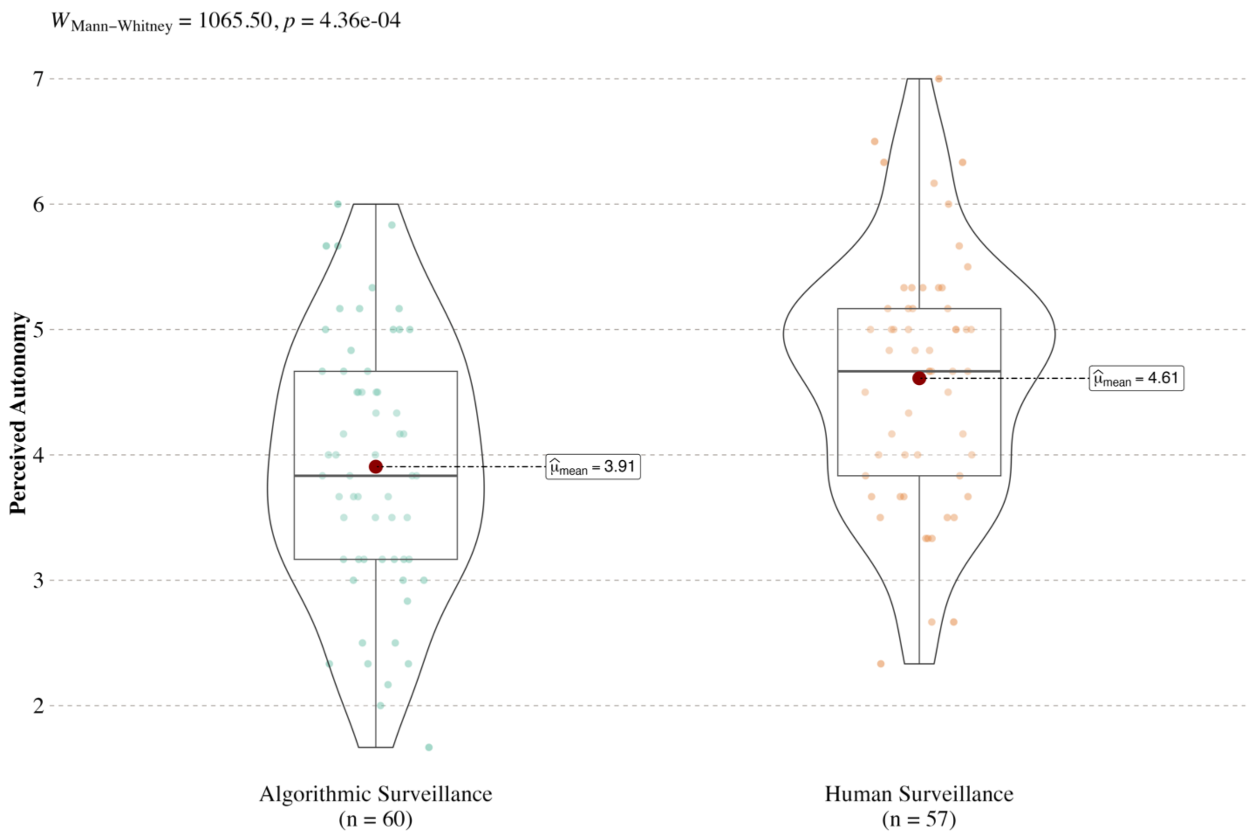

Supplementary Figure 6

Perceived Autonomy by Condition in Study 4.

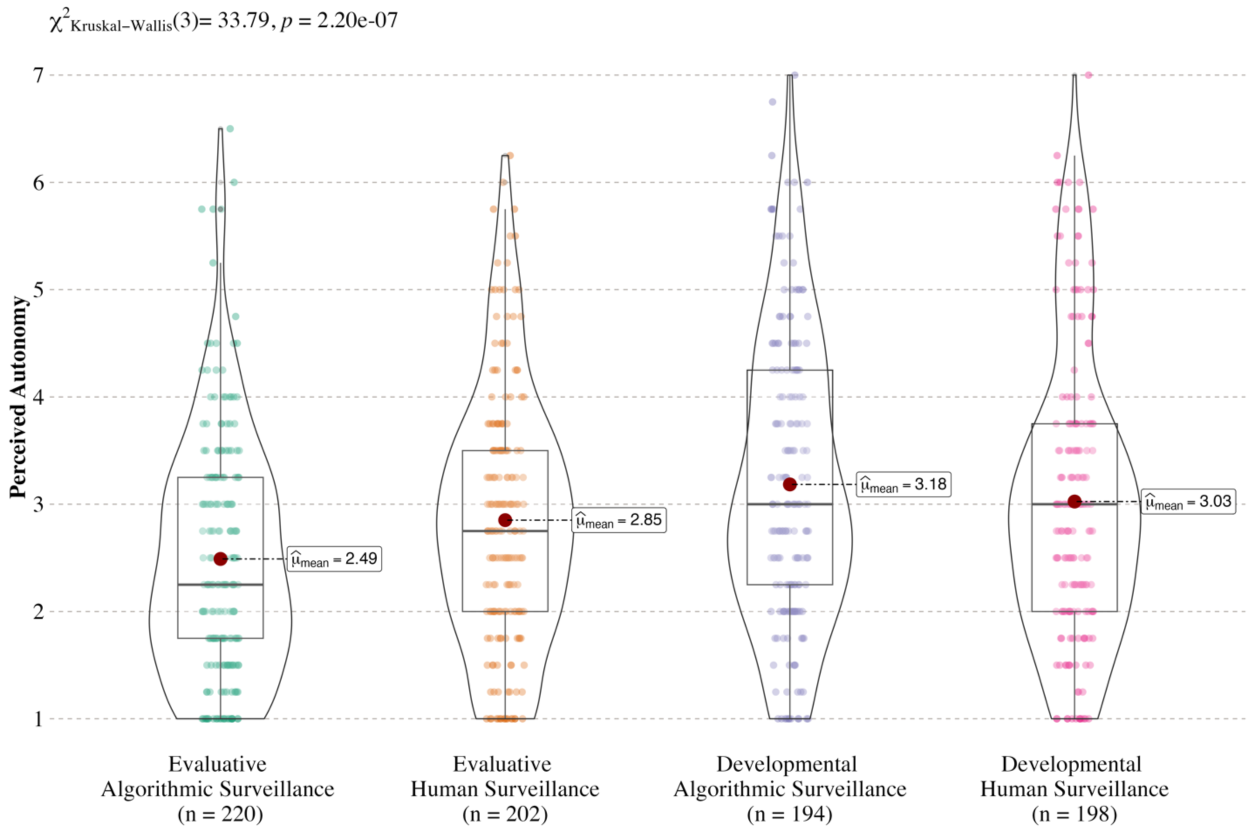

Supplementary Figure 7

Resistance Intentions by Condition in Study 4.

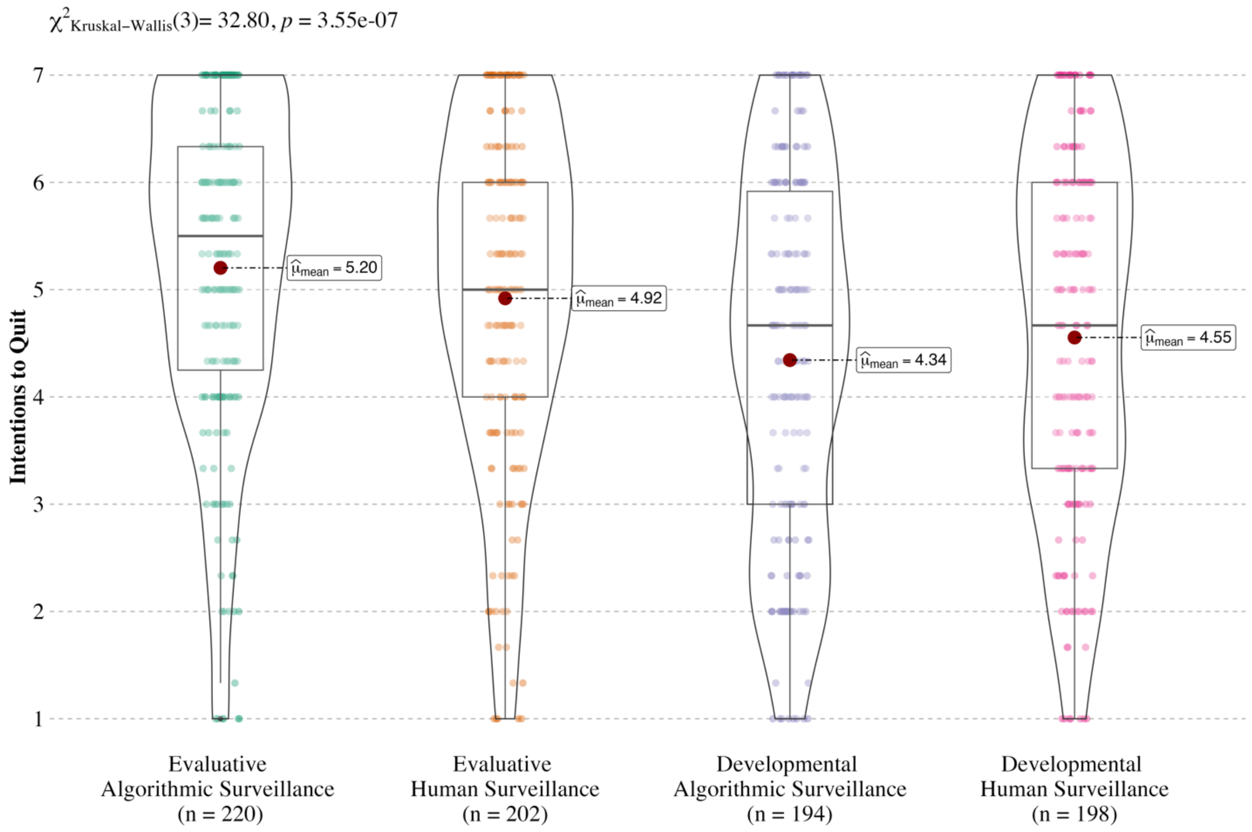

Supplement: Supplementary file 2 — Supplementary Information [file 44271_2024_102_MOESM2_ESM.pdf]
