# Supplementary material for: Algorithmic versus human surveillance leads to lower perceptions of autonomy and increased resistance
Source: Commun Psychol. 2024 Jun 6;2:53. doi: 10.1038/s44271-024-00102-8 (PMC11332184; doi:10.1038/s44271-024-00102-8)
Supplement: Supplementary file 3 — Reporting Summary [file 44271_2024_102_MOESM3_ESM.pdf]

Corresponding author(s): Rachel Schlund

Last updated by author(s): 24.03.06

Reporting Summary

Nature Portfolio wishes to improve the reproducibility of the work that we publish. This form provides structure for consistency and transparency in reporting. For further information on Nature Portfolio policies, see our Editorial Policies and the Editorial Policy Checklist.

Statistics

For all statistical analyses, confirm that the following items are present in the figure legend, table legend, main text, or Methods section.

n/a	Confirmed	
☐	☒	The exact sample size (*n*) for each experimental group/condition, given as a discrete number and unit of measurement
☐	☒	A statement on whether measurements were taken from distinct samples or whether the same sample was measured repeatedly
☐	☒	The statistical test(s) used AND whether they are one- or two-sided<br>*Only common tests should be described solely by name; describe more complex techniques in the Methods section.*
☐	☒	A description of all covariates tested
☐	☒	A description of any assumptions or corrections, such as tests of normality and adjustment for multiple comparisons
☐	☒	A full description of the statistical parameters including central tendency (e.g. means) or other basic estimates (e.g. regression coefficient) AND variation (e.g. standard deviation) or associated estimates of uncertainty (e.g. confidence intervals)
☐	☒	For null hypothesis testing, the test statistic (e.g. *F*, *t*, *r*) with confidence intervals, effect sizes, degrees of freedom and *P* value noted<br>*Give P values as exact values whenever suitable.*
☒	☐	For Bayesian analysis, information on the choice of priors and Markov chain Monte Carlo settings
☐	☒	For hierarchical and complex designs, identification of the appropriate level for tests and full reporting of outcomes
☐	☒	Estimates of effect sizes (e.g. Cohen's *d*, Pearson's *r*), indicating how they were calculated

Our web collection on statistics for biologists contains articles on many of the points above.

Software and code

Policy information about availability of computer code

Data collection	University lab, Cloud Research, Qulatrics
Data analysis	SPSS and R

For manuscripts utilizing custom algorithms or software that are central to the research but not yet described in published literature, software must be made available to editors and reviewers. We strongly encourage code deposition in a community repository (e.g. GitHub). See the Nature Portfolio guidelines for submitting code & software for further information.

Data

Policy information about availability of data

All manuscripts must include a data availability statement. This statement should provide the following information, where applicable:

- Accession codes, unique identifiers, or web links for publicly available datasets
- A description of any restrictions on data availability
- For clinical datasets or third party data, please ensure that the statement adheres to our policy

All data and materials are available on the Open Science Framework (https://osf.io/3ztpm/?view_only=91c58da2216f4633b98d5fa1cb849808), and pre-registrations can be found on aspredicted.org.

Research involving human participants, their data, or biological material

Policy information about studies with human participants or human data. See also policy information about sex, gender (identity/presentation), and sexual orientation and race, ethnicity and racism.

Reporting on sex and gender	Study 1. 50.5% identified as women; 47.7% as men; 1.8% as non-binary/gender non-conforming.<br><br>Study 2. 54.8% identified as women; 45.2% as men.<br><br>Study 3. 70% identified as women; 25.6% as men; .9% as cisgender men; 1.8% as cisgender women; .9% as non-binary/gender non-conforming; .9% did not report on their gender.<br><br>Study 4. 58.8% identified as women; 39.2% as men; .8% as non-binary/gender non-conforming; .2% as agender; .1% as cisgender women; .1% as cisgender men; .2% as transmasculine; .6% did not report on their gender.
Reporting on race, ethnicity, or other socially relevant groupings	Study 1. 11.2% as Black/African American; 6.5% as Asian/Asian American/Pacific Islander; 70.1% as White/European American; 1.9% as Latino/Hispanic American; 1.9% as Middle Eastern/Arab American; and 8.4% as Biracial/Mixed-Race<br><br>Study 2. 5.1% as Black/African American; 17.2% as Asian/Asian American/Pacific Islander; 45.2% as White/European American; 7.6% as Latino/Hispanic American; 3.2% as Middle Eastern/Arab American; and 21.7% as Biracial/Mixed-Race<br><br>Study 3. 5.1% as Black/African American; 38.5% as Asian/Asian American/Pacific Islander; 39.3% as White/European American; 1.7% as Latino/Hispanic American; .9% as Middle Eastern/Arab American; and 13.6% as Biracial/Mixed-Race; .9% did not report on their race/ethnicity<br><br>Study 4. 10.3% as Black/African American; 6.3% as Asian/Asian American/Pacific Islander; 71.9% as White/European American; 4.8% as Latino/Hispanic American; .2% as Middle Eastern/Arab American; .1% as Native American; 6.3% as Biracial/Mixed-Race; and .1% did not report on their race
Population characteristics	Study 1. 50.5% identified as women; 47.7% as men; 1.8% as non-binary/gender non-conforming; 11.2% as Black/African American; 6.5% as Asian/Asian American/Pacific Islander; 70.1% as White/European American; 1.9% as Latino/Hispanic American; 1.9% as Middle Eastern/Arab American; and 8.4% as Biracial/Mixed-Race; Mage = 37.09, SDage= 11.06<br><br>Study 2. 54.8% identified as women; 45.2% as men; 5.1% as Black/African American; 17.2% as Asian/Asian American/Pacific Islander; 45.2% as White/European American; 7.6% as Latino/Hispanic American; 3.2% as Middle Eastern/Arab American; and 21.7% as Biracial/Mixed-Race; Mage = 18.57, SDage= 1.17<br><br>Study 3. 70% identified as women; 25.6% as men; .9% as cisgender men; 1.8% as cisgender women; .9% as non-binary/gender non-conforming; .9% did not report on their gender; 5.1% as Black/African American; 38.5% as Asian/Asian American/Pacific Islander; 39.3% as White/European American; 1.7% as Latino/Hispanic American; .9% as Middle Eastern/Arab American; and 13.6% as Biracial/Mixed-Race; .9% did not report on their race/ethnicity; Mage = 22.57, SDage= 8.11<br><br>Study 4. 58.8% identified as women; 39.2% as men; .8% as non-binary/gender non-conforming; .2% as agender; .1% as cisgender women; .1% as cisgender men; .2% as transmasculine; .6% did not report on their gender; 10.3% as Black/African American; 6.3% as Asian/Asian American/Pacific Islander; 71.9% as White/European American; 4.8% as Latino/Hispanic American; .2% as Middle Eastern/Arab American; .1% as Native American; 6.3% as Biracial/Mixed-Race; and .1% did not report on their race; Mage = 39.99, SDage= 12.25
Recruitment	All participants were convenience samples. Participants in Studies 1 and 4 were recruited from online knowledge platforms (i.e., CloudResearch), while the participants in Studies 2 and 3 were recruited from a large university laboratory.
Ethics oversight	The Institutional Review Board at Cornell University

Note that full information on the approval of the study protocol must also be provided in the manuscript.

Field-specific reporting

Please select the one below that is the best fit for your research. If you are not sure, read the appropriate sections before making your selection.

☐ Life sciences ☒ Behavioural & social sciences ☐ Ecological, evolutionary & environmental sciences

For a reference copy of the document with all sections, see nature.com/documents/nr-reporting-summary-flat.pdf

Life sciences study design

All studies must disclose on these points even when the disclosure is negative.

Sample size	NA

Data exclusions	NA
Replication	NA
Randomization	NA
Blinding	NA

Behavioural & social sciences study design

All studies must disclose on these points even when the disclosure is negative.

Study description	All studies utilize quantitative experimental designs.
Research sample	Study 1. The final sample included 107 participants.<br>Study 2. The final sample included 157 participants.<br>Study 3. The final sample included 117 participants.<br>Study 4. The final sample included 814 participants.
Sampling strategy	Study 1. We recruited 120 participants. We determined this sample size based on the effect size we obtained in a pilot study, our desire for 80% power, and our expected number of exclusions.<br><br>Study 2. Since it was not possible to determine exactly how many people would choose to participate, we aimed to collect as many participants as possible within a pre-determined time period, with the goal of recruiting at least 50 participants per cell (Simmons et al., 2011).<br><br>Study 3. Since it was not possible to determine exactly how many people would choose to participate, we aimed to collect as many participants as possible within a pre-registered, pre-determined time period, with the goal of recruiting at least 50 participants per cell (Simmons et al., 2011).<br><br>Study 4. We recruited 814 participants via Cloud Research, based on our recruiting heuristic of 200 participants per cell.
Data collection	Studies 1 and 4 utilized Qualtrics surveys that were delivered electronically. Studies 2 and 3 were conducted at a university lab and the questionnaires were collected using Qualtrics.
Timing	Study 1. November 2023<br><br>Study 2. November 2020<br><br>Study 3. May 2021<br><br>Study 4. November 2022
Data exclusions	Study 1. As pre-registered, participants were required to pass a simple CAPTCHA check in order to gain access to the survey, with many of those screened out likely being bots. Also, as pre-registered, we excluded participants who failed to recall an event, who described an event that was off-topic, or who wrote a response that was not interpretable.<br><br>Study 2. No participants were excluded from the analysis.<br><br>Study 3. During this study, the technology failed twice, preventing some participants from going through the full experimental procedure. We excluded their data (n=10).<br><br>Study 4. As pre-registered, participants were required to pass a CAPTCHA check in order to gain access to the survey, with many of those screened out likely being bots.
Non-participation	No participant declined participation.
Randomization	Study 1 utilized a within-subjects design, but the order of the manipulations was randomized to avoid potential order effects. In Studies 2 and 3 participants were randomly assigned to one of the two experimental conditions. In Study 4, participants were randomly assigned to one of the four experimental conditions.

Ecological, evolutionary & environmental sciences study design

All studies must disclose on these points even when the disclosure is negative.

Study description	NA

Research sample	NA
Sampling strategy	NA
Data collection	NA
Timing and spatial scale	NA
Data exclusions	NA
Reproducibility	NA
Randomization	NA
Blinding	NA

Did the study involve field work? ☐ Yes ☐ No

Field work, collection and transport

Field conditions	NA
Location	NA
Access & import/export	NA
Disturbance	NA

Reporting for specific materials, systems and methods

We require information from authors about some types of materials, experimental systems and methods used in many studies. Here, indicate whether each material, system or method listed is relevant to your study. If you are not sure if a list item applies to your research, read the appropriate section before selecting a response.

Materials & experimental systems

n/a	Involved in the study
☒	☐ Antibodies
☒	☐ Eukaryotic cell lines
☒	☐ Palaeontology and archaeology
☒	☐ Animals and other organisms
☒	☐ Clinical data
☒	☐ Dual use research of concern
☒	☐ Plants

Methods

n/a	Involved in the study
☒	☐ ChIP-seq
☒	☐ Flow cytometry
☒	☐ MRI-based neuroimaging

Antibodies

Antibodies used	NA
Validation	NA

Eukaryotic cell lines

Policy information about cell lines and Sex and Gender in Research

Cell line source(s)	NA
Authentication	NA
Mycoplasma contamination	NA

Commonly misidentified lines (See ICLAC register)	NA

Palaeontology and Archaeology

Specimen provenance	NA
Specimen deposition	NA
Dating methods	NA

☐ Tick this box to confirm that the raw and calibrated dates are available in the paper or in Supplementary Information.

Ethics oversight	NA

Note that full information on the approval of the study protocol must also be provided in the manuscript.

Animals and other research organisms

Policy information about studies involving animals; ARRIVE guidelines recommended for reporting animal research, and Sex and Gender in Research

Laboratory animals	NA
Wild animals	NA
Reporting on sex	NA
Field-collected samples	NA
Ethics oversight	NA

Note that full information on the approval of the study protocol must also be provided in the manuscript.

Clinical data

Policy information about clinical studies

All manuscripts should comply with the ICMJE guidelines for publication of clinical research and a completed CONSORT checklist must be included with all submissions.

Clinical trial registration	NA
Study protocol	NA
Data collection	NA
Outcomes	NA

Dual use research of concern

Policy information about dual use research of concern

Hazards

Could the accidental, deliberate or reckless misuse of agents or technologies generated in the work, or the application of information presented in the manuscript, pose a threat to:

No	Yes	
☒	☐	Public health
☒	☐	National security
☒	☐	Crops and/or livestock
☒	☐	Ecosystems
☒	☐	Any other significant area

Experiments of concern

Does the work involve any of these experiments of concern:

No	Yes	
☒	☐	Demonstrate how to render a vaccine ineffective
☒	☐	Confer resistance to therapeutically useful antibiotics or antiviral agents
☒	☐	Enhance the virulence of a pathogen or render a nonpathogen virulent
☒	☐	Increase transmissibility of a pathogen
☒	☐	Alter the host range of a pathogen
☒	☐	Enable evasion of diagnostic/detection modalities
☒	☐	Enable the weaponization of a biological agent or toxin
☒	☐	Any other potentially harmful combination of experiments and agents

Plants

Seed stocks	NA
Novel plant genotypes	NA
Authentication	NA

ChIP-seq

Data deposition

☐ Confirm that both raw and final processed data have been deposited in a public database such as GEO.

☐ Confirm that you have deposited or provided access to graph files (e.g. BED files) for the called peaks.

Data access links<br>*May remain private before publication.*	NA
Files in database submission	NA
Genome browser session<br>(e.g. UCSC)	NA

Methodology

Replicates	NA
Sequencing depth	NA
Antibodies	NA
Peak calling parameters	NA
Data quality	NA
Software	NA

Flow Cytometry

Plots

Confirm that:

☐ The axis labels state the marker and fluorochrome used (e.g. CD4-FITC).

☐ The axis scales are clearly visible. Include numbers along axes only for bottom left plot of group (a 'group' is an analysis of identical markers).

☐ All plots are contour plots with outliers or pseudocolor plots.

☐ A numerical value for number of cells or percentage (with statistics) is provided.

Methodology

Sample preparation	NA
Instrument	NA
Software	NA
Cell population abundance	NA
Gating strategy	NA

☐ Tick this box to confirm that a figure exemplifying the gating strategy is provided in the Supplementary Information.

Magnetic resonance imaging

Experimental design

Design type	NA
Design specifications	NA
Behavioral performance measures	NA

Acquisition

Imaging type(s)	NA
Field strength	NA
Sequence & imaging parameters	NA
Area of acquisition	NA

Diffusion MRI ☐ Used ☐ Not used

Preprocessing

Preprocessing software	NA
Normalization	NA
Normalization template	NA
Noise and artifact removal	NA
Volume censoring	NA

Statistical modeling & inference

Model type and settings	NA
Effect(s) tested	NA

Specify type of analysis: ☐ Whole brain ☐ ROI-based ☐ Both

Statistic type for inference (See Eklund et al. 2016)	NA
Correction	NA

Models & analysis

n/a | Involved in the study

- [ ] [ ] Functional and/or effective connectivity
- [ ] [ ] Graph analysis
- [ ] [ ] Multivariate modeling or predictive analysis

Functional and/or effective connectivity	NA
Graph analysis	NA
Multivariate modeling and predictive analysis	NA
